# Positioning blinatumomab in the frontline of adult B-cell acute lymphoblastic leukemia treatment

**DOI:** 10.3389/fonc.2023.1237031

**Published:** 2023-08-17

**Authors:** Hoda Pourhassan, Vaibhav Agrawal, Vinod Pullarkat, Ibrahim Aldoss

**Affiliations:** Department of Hematology and Hematopoietic Cell Transplantation, Gehr Family Center for Leukemia Research, City of Hope National Medical Center, Duarte, CA, United States

**Keywords:** blinatumomab, frontline, acute lymhoblastic leukemia, adult, B cell

## Abstract

Blinatumomab is a bispecific T cell engager that has shown efficacy in relapsed/refractory Philadelphia chromosome (Ph)-positive and Ph-negative acute lymphoblastic leukemia (ALL). Considering its favorable safety and activity in advanced ALL, blinatumomab as a targeted immunotherapy is fast gaining a frontline position in the ALL treatment paradigm. There have been multiple completed and ongoing studies showing significant promise with improved response rates and survival outcomes and decreased treatment toxicity and need for multi-agent chemotherapy regimens. The early use of blinatumomab has established success in Ph-negative and Ph-positive B-ALL, and this has extended to older adults with ALL who have historically had substantially inferior outcomes compared to their pediatric and young adult counterparts. Herein we will review the current data describing the early use of blinatumomab in newly diagnosed adults with B-cell ALL and future directions.

## Introduction

The treatment of adult B-cell acute lymphoblastic leukemia (ALL) has witnessed substantial evolution over the recent years. With increasing understanding of the molecular and genetic blueprint of ALL, we are able to better tailor treatment regimens and thereby improve outcomes. The development of effective salvage therapies has led to improved outcomes of relapsed/refractory (r/r) B-ALL, and these novel drugs are being advanced to frontline therapies in ongoing clinical trials. Blinatumomab is a bispecific T cell engager that binds to CD19 antigen on B-lymphoblasts and CD3 on T cells, leading to the activation and proliferation of T cells that can exert cytotoxic effects against CD19-positive leukemic cells (1). Blinatumomab has shown encouraging efficacy in the r/r setting, including measurable residual disease (MRD) relapse (2). In r/r Ph-negative (Ph-) B-ALL, treatment with blinatumomab has resulted in superior response rate and median overall survival (OS) when compared to chemotherapy (3–5). In the case of Ph- positive (Ph+) ALL, blinatumomab produced promising complete remission (CR) rates in tyrosine kinase inhibitor (TKI)-refractory patients and demonstrated an ability to overcome the adverse impact of high-risk mutations, including *T315I* (6). Blinatumomab has a relatively favorable safety profile when compared to chemotherapy, and this is specifically relevant to the older patient population where toxicity can be a limitation for administering curative chemotherapy regimens (7). The optimal utilization of blinatumomab in B-cell ALL has continued to evolve, and its use is advancing to the frontline setting with the intent to enhance the depth of remissions, prevent relapses, and allow for de-escalation of chemotherapy regimens in order to reduce toxicity and improve outcomes. Herein we will discuss the early use of blinatumomab in newly diagnosed B-cell ALL.

## Frontline blinatumomab in younger adults with Ph- negative ALL

The outcomes of younger adult patients with newly diagnosed Ph- ALL have improved with the adoption of pediatric-inspired regimens and better disease-risk stratification based on MRD response and disease genomics (8–11). Nonetheless, adults with Ph- ALL continue to have inferior outcomes compared to their pediatric counterparts as the result of higher frequencies of adverse-risk genetics and toxicities experienced with regimens administered with curative intent (12, 13). Several groups have therefore investigated blinatumomab as frontline therapy for young adults with Ph- ALL and have aimed to optimize its use by incorporating it at different stages of treatment.

The GIMEMA LAL2317 trial (14) evaluated 146 patients aged 18–65 and incorporated two blinatumomab cycles given sequentially after early consolidation “cycle 3” and late consolidation “cycle 6”, respectively. Hematological CR was achieved in 90% of patients following induction. The primary objective was MRD- rate following the first blinatumomab cycle, which was attained in 96% of patients with a conversion rate from MRD+ to MRD- of 87%. The 12-month OS and disease-free survival (DFS) rates were 84% and 72%, respectively, demonstrating not only the efficacy of blinatumomab added to chemotherapy in increasing MRD negativity but also a lower early relapse rate. However, these findings were preliminary and based on limited follow-up.

The GRAALL-2014 trial (15) focused specifically on high-risk patients identified by the presence of *KMT2A* rearrangements, *IKZF1* intragenic deletion (*IKZF1*del), or persistent MRD following induction (16). A total of 95 patients aged 18–60 years in continuous CR after induction and consolidation 1 were prospectively identified and enrolled to receive blinatumomab starting at week 12. Patients with indication for allogeneic hematopoietic stem cell transplantation (alloHSCT) received blinatumomab for a minimum of 4 weeks prior to proceeding to transplant, while all other patients received five cycles during the consolidation and maintenance phases. After blinatumomab therapy, MRD negativity was achieved in 74% of evaluable patients with pre-blinatumomab MRD. The MRD response to blinatumomab was lower in patients with a higher pretreatment MRD level, while it was not impacted by age, initial white blood cell count, or genetic subgroup. The 18-month DFS and OS were 79% and 92%, respectively, demonstrating the encouraging efficacy of early blinatumomab administration during consolidation in the high-risk population.

The concept of early blinatumomab use has also been implemented with the intent to decrease the chemotherapy intensity and burden. A phase 2 Australasian Leukemia and Lymphoma Group (ALLG) study (17) evaluated reduced-intensity chemotherapy in combination with blinatumomab. Patients received debulking low-intensity chemotherapy with cyclophosphamide, vincristine, and dexamethasone, followed by 7 days of blinatumomab. The patients then received three alternating cycles of blinatumomab and part B cycles of hyper-CVAD, followed by 2 years of maintenance therapy in subjects not proceeding to alloHSCT. All patients attained a CR, and of 26 patients evaluable for MRD, 70% had achieved an MRD response after cycle 1B and 83% at the end of cycle 2B. The estimated 24-month event-free survival (EFS) and OS were 62% and 69%, respectively. Overall, the combination of blinatumomab with chemotherapy was tolerable and efficacious with high rates of CR and MRD response.

A single-arm, phase 2 trial conducted at MD Anderson Cancer Center (MDACC) (18) evaluated 38 patients aged 14 and older with B-ALL. The treatment consisted of 4 cycles of intensive chemotherapy with hyper-CVAD, followed by 4 cycles of blinatumomab consolidation and then maintenance therapy with alternating blocks of POMP and blinatumomab for a total of 15 treatment cycles. The estimated 3-year relapse-free survival (RFS) was 73%, and no patient relapsed more than 2 years after starting the therapy. This study was later amended to incorporate the targeted CD22–antibody–drug conjugate inotuzumab ozogamicin (InO) (19) which was added sequentially to 2 cycles of methotrexate/cytarabine and to 2 cycles of blinatumomab. Among patients with active disease at study entry, all achieved CR, including 76% achieving MRD- status after induction and 95% at any time during therapy. Overall, 9% of patients relapsed, 31% underwent transplant in first remission, and 57% remained in remission without transplant. It is also notable that all relapses occurred in patients with established poor-risk features. For the entire cohort, the estimated 3-year OS was 85%, and the 3-year continuous remission duration was 84%. No relapses nor deaths occurred in the InO group, and the estimated 1-year OS was 100%.

In the phase 3 ECOG-1910 trial, 224 adult patients with B-ALL who achieved MRD- CR after intensification were randomized to conventional chemotherapy with or without blinatumomab (20). The patients initially received 2.5 months of induction chemotherapy utilizing a BFM-like regimen with extended remission induction. Pegaspargase was added for patients <55 years of age. After remission induction, if the patients were in CR they continued on the study and received an intensification course of high-dose methotrexate with pegaspargase, after which remission and MRD status were determined. All patients in MRD- CR were then randomized to receive an additional 4 cycles of consolidation chemotherapy or four alternating cycles of blinatumomab and consolidation chemotherapy. The addition of blinatumomab to consolidation chemotherapy resulted in a significantly better median OS (not reached vs. 71 months). Death due to ALL and death in remission were lower in the blinatumomab arm compared to the chemotherapy arm. No safety concerns were noted with alternating blinatumomab and chemotherapy during consolidation.

These results show the promise of utilizing blinatumomab in combination with intensive chemotherapy early in the course of treatment of younger adults and may represent a new standard of care in Ph- negative adult ALL. **Table 1** summarizes the published studies of frontline blinatumomab use in newly diagnosed young adults with Ph- B-ALL.

**Table 1:** Blinatumomab studies in the frontline treatment of Ph-negative acute lymphoblastic leukemia.

Study	Blinatumomab design	Number of patients	Median age (range)	Grade ≥3 CRS	Grade ≥3 neurotoxicity	CR rate	MRD negative rate of evaluable patients	Induction death	OS	EFS/RFS/DFS
A. Young adults										
**GIMEMA LAL2317**	Sequential blinatumomab following early and late consolidation	146	41 years (18-65 years)	NA	NA	90.4%	73% (after early consolidation) 96% (following first blinatumomab)	NA	83.8% (12 months)	DFS 71.6% (12 months)
**GRAALL-2014**	Consolidation and maintenance blinatumomab	95	35 years (18–60 years)	0%	6%	NA	74%	NA	92.1% (18 months)	DFS 68.8% (18 months)
**ALLG ALL08 Study**	Low-intensity chemotherapy followed by blinatumomab	30	51.7 years (39.5–66.5 years)	3%	NA	100% by end of 2B	70% (end of cycle 1B) (83% end of cycle 2B)	0	68.65 (24 months)	EFS 61.8% (24 months)
**MDACC, Jabbour et al. (Blina cohort)**	High-dose induction chemotherapy followed by blinatumomab consolidation	38	37 years (17–59 years)	3%	11%	100%	(76% following induction) (95% at any time over the therapy course)	NA	85% (3 years)	RFS 84% (3 years)
**MDACC, Short et al. (Blina + Ino cohort)**	Amendment to Jabbour et al. with the addition of inotuzumab	20	24 (18–47 years)							
**ECOG-1910**	Conventional chemotherapy +/- blinatumomab	488	51 years (30–70 years)	NA	NA	81%	NA	NA	Median OS: not reached (71.4 months)	NA
B. Older adults										
**SWOG 1318**	Single-agent blinatumomab induction and consolidation followed by chemotherapy maintenance	29	75 years (NA)	3%	3%	66%	NA	NA	37% (3 years)	DFS 37% (3 years)
**MDACC, Kantarjian et al.**	Inotuzumab and chemotherapy induction with blinatumomab consolidation and maintenance	64	68 years (60–81 years)	NA	NA	98%	77% (after 1 cycle) 94% (overall)	0% (30-day mortality) 3% (60-day mortality)	54% (overall; 3 years) 42% (≥70 years; 3 years) 63% (60–69 years; 3 years)	Continuous remission: 76% (overall; 3 years) 87% (≥70 years; 3 years) 69% (60–69 years; 3 years)
**GMALL Bold Trial**	Blinatumomab in sequence with induction chemotherapy	33	65 years (56–76 years)	NA	NA	76% (after induction 1) 83% (after blina 1)	NA	6%	100% (55–65 years; 1 year) 66% (>65 years; 1 year)	DFS 89% (1 year)
**ALLIANCE A041703 Study**	Sequential inotuzumab induction and blinatumomab consolidation	33	71 years (60–84 years)	NA	NA	85% (induction IA/B/C) 97% (course II)	NA	NA	84% (1 year)	EFS 75% (1 year)
**Golden Gate Study**	Blinatumomab alternating with low-intensity induction chemotherapy	10	69 years (57–77 years)	0%	0%	100%	90% after induction cycle 1 100% after induction cycle 2	0%	NA	NA

CR, complete remission; DFS, disease-free survival; EFS, event-free survival; MRD, minimal residual disease; NA, not available; Ph, Philadelphia chromosome; RFS, relapse-free survival.

## Frontline blinatumomab in older adults with Ph- negative ALL

Historically, older adults with Ph- ALL had dismal outcomes with chemotherapy treatment with a 5-year OS of 10–20% (21). This is attributable to both the higher incidence of adverse genetic findings as well as poor tolerance to chemotherapy regimens (21, 22). Even with age-adapted, dose-reduced regimens, early death rates remain considerably high (23). These observations have rationalized the introduction of blinatumomab in the frontline treatment paradigm for older adults with B-ALL.

In the SWOG 1318 study conducted in patients aged ≥65 years (24), single-agent blinatumomab was implemented as an induction therapy for 1 to 2 cycles until the attainment of response, followed by 3 cycles of consolidation with blinatumomab and subsequently 18 months of maintenance chemotherapy. The study enrolled 29 eligible patients and demonstrated a CR rate of 66%. The 3-year DFS and OS estimates were 37% and 37%, which compared favorably to historical studies for this age group.

The phase II study by Kantarjian et al. (25) extrapolated from their initial finding that combining InO with reduced-intensity mini-hyper-CVD chemotherapy is both safe and highly effective (26) and incorporated blinatumomab as a consolidation therapy to further improve the outcomes. The study enrolled 64 patients ≥60 years who received 4 cycles of hyper mini-CVD plus InO, followed by 4 cycles of blinatumomab and then maintenance with 1 cycle of blinatumomab after every 3 cycles of POMP for a total of 12 cycles. In total, 98% of patients attained CR, and the MRD- rates were 77% after the first cycle and 94% anytime during therapy. There were no early deaths, and the 60-day mortality rate was 3%. The 3-year continuous remission rate and OS were 76% and 54%, respectively. When the patients were stratified by age, the 3-year continuous CR rates were 69% for patients age 60–69 and 87% for those ≥70 years, while the 3-year OS rates were 63% and 42%, respectively. The inferior survival in the patients ≥70 years was driven predominantly by the increased rate of death in CR. The outcomes of patients who did or did not receive blinatumomab were surprisingly similar. These results confirmed that reduced-intensity chemotherapy with hyper mini-CVD plus InO, with or without blinatumomab, is safe and effective in older adults with newly diagnosed Ph- ALL.

A trial by the GMALL study group evaluated blinatumomab administered sequentially with chemotherapy among patients aged 56–76 years with B-ALL (23). In this study, patients with complete or partial response to initial chemotherapy received blinatumomab, while those with induction failure were treated with a second induction cycle, followed by blinatumomab. The consolidation treatment consisted of three further cycles of blinatumomab and then standard maintenance up to a total treatment duration of 2 years. Of 33 patients evaluable after the initial induction phase, 85% responded, 9% had treatment failure, and 29% had a molecular response. One-third of patients with failure after initial induction had a CR after second induction, resulting in 83% CR rate, and 82% of the CR patients achieved a molecular response following blinatumomab. OS was 100% at 1 year for patients aged 55–65 years and 66% for those older than 65. The 1-year DFS was 89% for all patients.

In the ALLIANCE A041703 study (27), patients aged ≥60 years old with Ph-, CD22+ (≥20% of lymphoblasts) B-ALL with no plan for transplant were treated with sequential InO induction, followed by blinatumomab consolidation. Induction IA consisted of InO alone. Patients who achieved adequate cytoreduction (marrow blasts reduced by ≥50% or cellularity ≤20%) or CR/CRi were allocated to IB treatment cycle with InO. Patients who did not achieve CR/CRi were moved to IC treatment cycle with InO reinduction. If cytoreduction was not achieved after IA, these patients proceeded directly to course II with blinatumomab. Patients who achieved CR/CRi with InO received two more cycles of blinatumomab, while others received three additional cycles of blinatumomab. No maintenance was administered. Among 33 eligible patients, the cumulative CR rates through induction IA/B/C and course II were 85% and 97%, respectively. With a median follow-up of 22 months, the 1-year EFS was 75%.

Golden Gate is a phase 3 study enrolling newly diagnosed Ph- B-ALL in older adult patients aged ≥55 or 40 to <55 years with severe, pre-defined comorbidities. The study randomizes the patients to either blinatumomab alternating with low-intensity chemotherapy or standard chemotherapy. Patients randomized to the investigational arm received blinatumomab for 2 cycles during induction, 2 cycles during consolidation, and 3 cycles during maintenance therapy alternating with chemotherapy. In the safety run phase, 10 patients were treated on the investigational arm; the treatment was well tolerated, and no early deaths occurred. The regimen also proved efficacious as all patients enrolled achieved CR and 90% had an MRD response <10^-4^ after induction cycle 1 (28). The randomized phase of the study is actively enrolling patients.

Taken together, the results of these studies indicate favorable tolerability and efficacy for early blinatumomab use in older patients with B-ALL considering the high MRD response rate, low treatment-related mortality, and encouraging short-term survival outcomes. **Table 1** summarizes the published studies of frontline blinatumomab in older adults with Ph- B-ALL.

## Frontline blinatumomab in Ph+ ALL

Philadelphia chromosome (t9;22) is the most common cytogenetic abnormality among adult patients with ALL, accounting for 25% of cases, and its incidence increases in older adults (29). In the pre-tyrosine kinase inhibitor (TKI) era, patients with Ph+ ALL had dismal outcomes apart from those who were consolidated with alloHSCT in remission (30–32). Since the advent of TKIs, outcomes with Ph+ ALL have improved substantially across all ages, and studies have demonstrated that reducing the chemotherapy backbone has less impact on the treatment outcomes. This observation promoted the investigation of blinatumomab as a replacement of chemotherapy backbone in combination with TKIs in Ph+ ALL, which could potentially enhance treatment safety and efficacy.

In the LAL2116 (D-ALBA) trial (33), the GIMEMA group designed a chemotherapy-free regimen for newly diagnosed adults with Ph+ ALL (*n* = 63). The patients were induced with dasatinib and corticosteroid, followed by at least two cycles of blinatumomab with a maximum of 5 cycles. The post-consolidation treatment was left to the investigator’s choice. The molecular response rate was improved from 29% pre-blinatumomab to 60% after the first cycle of blinatumomab, and this increased further after additional cycles of blinatumomab. In an updated follow-up, the 24-month OS and DFS were 88% and 80%, respectively, and DFS was superior in patients achieving a complete molecular response (CMR) upon post-induction/consolidation compared to those who did not (100% vs. 75.9%, *p* = 0.028).

Advani et al. (34) implemented a similar regimen to the D-ALBA study but restricted the enrollment to older patients age ≥ 65 years with Ph+ ALL. For induction, the patients received dasatinib with prednisone, and those achieving CR continued to post-remission therapy with blinatumomab and dasatinib, while patients not achieving remission received re-induction with blinatumomab. This was followed by 3 cycles of post-remission therapy with blinatumomab and dasatinib, followed by maintenance with prednisone for 18 cycles and dasatinib indefinitely. Of the 25 patients accrued, 92% of patients achieved CR after induction and 31% of patients achieved CMR. The estimated 3-year OS and DFS rates were 85% and 80%, respectively. The treatment was well tolerated, with no early death or grade 4 or higher treatment-related non-hematologic toxicities.

A single-arm, phase 2 trial from MD Anderson Cancer Center was conducted in patients aged 18 years or older with newly diagnosed or r/r Ph-positive ALL or chronic myeloid leukemia in the lymphoid blast phase (35). This study implemented the chemotherapy-free regimen of ponatinib with blinatumomab for 5 cycles followed by ponatinib monotherapy. Of patients with newly diagnosed Ph+ ALL, 87% of the 38 evaluable patients had a CMR, and the estimated 2-year EFS and OS were both 95%. This chemotherapy-free regimen demonstrated a remarkable molecular response and very promising outcomes on short-term follow up, demonstrating that these patients could potentially be spared from the toxicities associated with chemotherapy and the need for alloHSCT in first CR.

Taken together, these chemotherapy-free strategies combining TKIs and blinatumomab in frontline treatment are highly effective and well tolerated in both young and older adults with Ph+ ALL. **Table 2** summarizes the studies incorporating blinatumomab in frontline regimens among adults with Ph+ ALL.

**Table 2:** Blinatumomab studies in frontline treatment of Ph-positive acute lymphoblastic leukemia.

Study	Blinatumomab design	Number of patients	Median age (range)	Grade ≥3 CRS	Grade ≥3 neurotoxicity	CR rate	MRD negative rate of evaluable patients	Induction death	OS	EFS/RFS/DFS
**LAL2116 (D-ALBA) trial**	Dasatinib plus steroids followed by blinatumomab consolidation	63	54 years (24–82 years)	NA	2%	98%	NA	NA	95% (18 months)	DFS 88% (18 months)
**Advani et al.**	Dasatinib plus steroids followed by post-remission blinatumomab and dasatinib	25	73 years (62–87 years)	NA	NA	92%	31%	0%	85% (3-year estimate)	DFS 80% (3-year estimate)
**MDACC, Jabbour et al.**	Ponatinib with blinatumomab induction followed by ponatinib monotherapy	72	51 years (36–68 years)	NA	NA	87%	NA	0%	NA	NA

CR, complete remission; DFS, disease-free survival; EFS, event-free survival; MRD, minimal residual disease; NA, not available; Ph, Philadelphia chromosome; RFS, relapse-free survival.

## The current and future state of blinatumomab in frontline regimens for adults with B-ALL

The frontline treatment paradigm for adult ALL is shifting rapidly. While previously limited to multiagent chemotherapy with poor tolerability and short lived remissions (31–33), we are now in a position to deescalate conventional chemotherapy regimens by incorporating novel targeted agents such as blinatumomab and InO. This strategy could be particularly relevant to older adults and could improve the dismal long-term survival observed in this age group.

It appears that initial debulking of disease with chemotherapy or TKI-based therapy (in cases of Ph+ ALL) provided the best setting for blinatumomab use at the present time. Studies in r/r B-ALL have established a correlation between leukemia disease burden and response to blinatumomab (36, 37). Administering blinatumomab in low disease burden is a safer strategy and has produced higher response and MRD-negativity (38), and among patients treated with blinatumomab for MRD+ disease, the responses can be durable (5).

In younger adults with Ph- ALL, consolidation with blinatumomab alternating with intensive chemotherapy is feasible and effective. Leukemia burden can be effectively cytoreduced with standard induction chemotherapy with or without InO, and blinatumomab is best to be delivered during consolidation cycles in the low- disease-burden state. The benefit of blinatumomab consolidation extends beyond patients in MRD+ CR1 and has been established even in patients in MRD-negative state. While the conversion of MRD+ to MRD- is high, the ECOG 1910 has demonstrated the survival advantage for blinatumomab when delivered to adults who achieved MRD- state with chemotherapy, and blinatumomab will likely become the new standard of care in this setting (20). Furthermore, alternating cycles of blinatumomab with intensive chemotherapy during consolidation is likely a more tolerable approach and enhances recovery from chemotherapy toxicity between cycles, even in young adults, as the ECOG study illustrated lower death rates among patients randomized to blinatumomab while in remission.

In older patients, blinatumomab is better positioned to be administered after cytoreduction therapy either with low-dose chemotherapy or InO. When administered in treatment-naïve patients with high disease burden in the SWOG 1813 study, CR/CRi was only 66% (24). In contrast, blinatumomab treatment was more tolerable and produced higher MRD- CR when it was delivered following low-intensity induction or cytoreduction therapy such as mini-hyper CVD + InO in MDACC (25), single-agent InO in the Alliance study (27), or low-dose chemotherapy in the Golden Gate study (28). In the MDACC study, the outcomes of older adults (>70 years) were inferior compared to those of younger patients, mainly driven by the excessive rate of death in remission and leading investigators to amend the study and eliminate chemotherapy in older patients. As studies have shown that blinatumomab in the r/r MRD+ ALL setting can be curative in some patients (5), the hope is that moving blinatumomab to the frontline consolidation setting will be a safe and curative therapeutic approach for older adults with B-ALL without relying on transplant as definitive consolidative therapy.

Finally, Ph+ disease represents a significant number of adults with B-ALL, especially older patients (21). The high activity of TKIs in this setting combined with the efficacy of blinatumomab may provide the opportunity to eliminate chemotherapy completely in this patient population. We are witnessing that the chemotherapy-free regimens of TKIs in conjunction with blinatumomab combination are becoming a reality in Ph+ ALL. However, there is a need to optimize which TKI to use and the timing of when to start blinatumomab. Data for the ponatinib–blinatumomab combination is very promising in this setting (35), but a longer follow-up and larger confirmatory studies are warranted. EA9181 (NCT04530565) is an ongoing phase 3 randomized study comparing TKI in combination with either blinatumomab or hyper-CVAD in adults with newly diagnosed Ph+ ALL, and the result of this study will likely transform the treatment paradigm of frontline therapy in Ph+ ALL.

Blinatumomab has a unique toxicity profile, and cytokine release syndrome (CRS) and neurotoxicity are the most common toxicities. A high disease burden was associated with an increased risk of CRS in r/r studies. In the frontline setting, the rates of high-grade CRS and neurotoxicity have varied by study design and the timing of blinatumomab. Grade 3/4 CRS was in 0%–3% but appeared lower in studies using blinatumomab later in the treatment course (15, 17, 18, 24, 28). In the case of neurotoxicity, grade 3/4 was reported in 0%–11% (15, 18, 24, 28, 33). Hence, initial debulking therapy prior to blinatumomab may minimize its toxicity.

Nonetheless, there are still several areas relevant to frontline blinatumomab use that warrant additional investigation. Identifying subsets of patients who are unlikely to respond is critical. The adverse impact of *IKZF1* deletion that was reported in Ph+ ALL patients treated with chemotherapy and TKI was also observed in the D-ALBA study with the use of blinatumomab and dasatinib (33). Additionally, high-risk genetics (*KMT2A* rearranged, low hypodiploidy/near triploidy, complex cytogenetics) predicted inferior outcomes in older adults with Ph- ALL treated with mini CVD + inotuzumab +/- blinatumomab (25). In Ph-like ALL, there has been an observed tendency for early relapse after blinatumomab treatment despite becoming MRD-negative. This was evidenced by a sub-analysis on a cohort of Ph-like ALL cases enrolled in the GIMEMA LAL2317 protocol and may call for a more targeted follow-up approach in these patients (39).

Extramedullary relapse, including the central nervous system (CNS), is an emerging concern with blinatumomab-based chemotherapy-free regimens (40). Given the lack of activity of blinatumomab against CNS diseases, particular attention should be provided towards optimizing CNS-directed therapy when chemotherapy is deescalated by the incorporation of blinatumomab — for instance, in the LAL2116 (D-ALBA) trial, patients treated with dasatinib and blinatumomab had a higher- than-expected proportion of CNS relapse, and this led the investigators of the LAL2820 Italian trial (NCT04722848) to increase the number of intrathecal chemotherapy doses in order to mitigate for this shortcoming (33, 41, 42).

Finally, the mode of administration of blinatumomab as continuous infusion can be a burden for patients and poses logistic challenges. Therefore, there has been interest in the alternate method of delivery by the subcutaneous route. The early experience with the subcutaneous (SC) formulation of blinatumomab is promising and merits further investigation. In the ongoing phase 1b dose-escalation study (NCT04521231), SC blinatumomab demonstrated an acceptable safety profile and encouraging anti-leukemia activity in heavily pretreated patients with r/r B-ALL (43). Data from 20 subjects have demonstrated pharmacokinetic exposures and pharmacodynamic profiles consistent with those reported for the intravenous formulation and boost the possibility for simplifying treatment administration and improving patient convenience.

The frontline of ALL treatment is arguably the most paramount line of defense as it will likely ultimately determine the chance of cure. Blinatumomab offers new hope, in the way of higher risk groups by age and molecular findings, that was previously out of reach and sets the stage for a much brighter future in ALL therapy.

## Author contributions

HP and IA completed the literature review and wrote and edited the manuscript. VP and VA edited the manuscript. All authors contributed to the article and approved the submitted version.
